# Investigating the physiological responses of Pacific white shrimp *Litopenaeus vannamei* to acute cold-stress

**DOI:** 10.7717/peerj.7381

**Published:** 2019-07-24

**Authors:** Zhenlu Wang, Yuexin Qu, Xiaolei Zhuo, Junyi Li, Jixing Zou, Lanfen Fan

**Affiliations:** 1 College of Marine Sciences, South China Agricultural University, Guangzhou, China; 2 Joint Laboratory of Guangdong Province and Hong Kong Region on Marine Bioresource Conservation and Exploitation, South China Agricultural University, Guangzhou, China; 3 Qingyuan North River Fishery Science Institute, Qingyuan, China

**Keywords:** *Litopenaeus vannamei*, Endoplasmic reticulum stress (ERS), Unfolded protein response (UPR), Cold stress

## Abstract

**Background:**

*Litopenaeus vannamei* is one of the most important aquaculture shrimps in the world and low temperatures present a serious challenge to its survival, growth, and distribution.

**Methods:**

To investigate their physiological responses during acute cold-stress, *L. vannamei* were treated under acute cooling conditions from 28 to 13 °C with a cooling rate of 2.5 °C/2 h and were maintained at 13 °C for 12 h. Plasma metabolite concentrations, histological changes, and relative gene expression related to the unfolded protein response (UPR) pathway and apoptosis in the hepatopancreas and the hemocytes of *L. vannamei* were investigated.

**Results:**

The results revealed that the concentrations of triglycerides, total cholesterol, and total protein in plasma reached their peaks at 23 °C, and then decreased to their minimum values at 13 °C for 12 h. The activity of alkaline phosphatase in the plasma decreased to its lowest level while the activity of alanine aminotransferase increased to its highest level at 13 °C for 12 h. The hepatic tubules became necrotic and the basement membranes were ruptured at 13 °C for 12 h. The gene expression related to UPR and apoptosis in the hepatopancreas and hemocytes was significantly altered by the decrease in the temperature.

**Discussion:**

The results revealed that acute cold-stress caused histological damage in the hepatopancreas of *L. vannamei*, reducing its immunity. The three UPR pathways were involved in the process of acute cold-stress and the response of activating transcription factor 6 to UPR may be faster and more directthan the *IRE1* and *PERK* pathways.

## Introduction

The pacific white shrimp, *Litopenaeus vannamei*, has become one of the most important aquaculture shrimps in the world due to a number of characteristics making it suitable for intensive aquaculture, including a wide degree of salt tolerance and rapid growth. However, a variety of environmental stimuli affect the growth of shrimp, especially temperature, which is highly influential and an annual cold wave causes huge economic losses to the *L. vannamei* breeding industry. Therefore, it is important to investigate the physiological responses of *L. vannamei* to stress caused by low temperatures.

Acute temperature changes may lead to arrests in growth or even death. Previous studies have shown that the genes involved in energy release and the biosynthesis of unsaturated fatty acids were increased with low temperature stress in *Pinctada fucata* (Wang et al., 2018). Low temperatures caused increases in dopamine and norepinephrine, as well as significant oxidative and antioxidant responses in *L. vannamei* (Mapanao et al., 2018; Xu et al., 2018). At 13 °C, *L. vannamei* has significant reductions in feeding and swimming, and deaths were noted at this temperature (Fan, Wang & Wu, 2013; Huang et al., 2017). Our previous study has indicated that *GRP78*, which is a central regulator of the process of the unfolded protein response (UPR) and apoptosis in the endoplasmic reticulum (ER) (Dejean, Martinez-caballero & Kinnally, 2006; Nakka, Gusain & Raghubir, 2010), was significantly up-regulated in the hepatopancreas of *L. vannamei* under 13 °C for 24 h post cold stress (Fan et al., 2016).

In invertebrates, UPR is generally recognized as a key mechanism of the endoplasmic reticulum stress (ERS) response (Chen & He, 2019). UPR includes three classic signaling pathways: the activating transcription factor 6 (*ATF6*) pathway, the inositol-requiring enzyme-1-X-box binding protein 1 (*IRE1-XBP1*) pathway, and the protein kinase RNA (*PKR*)-like ER kinase-*eIF2α* (*PERK-eIF2α*) pathway (Sidrauski & Walter, 1997; Mori, 2009; Costa, Rosa & De Camargo, 2011). The *ATF6* and *IRE1*-XBP1 pathways help to mitigate imbalances by increasing the capacity for proper protein folding (Li et al., 2000; Chakrabarti, Chen & Varner, 2011; Sun et al., 2015), while the *PERK*-*eIF2α* pathway can alleviate ERS by decreasing the load of newly synthesized peptides (Harding et al., 2000; Kebache et al., 2004). UPR is a self-protective mechanism and induces rescue or an adaptive response. However, if stress is prolonged, apoptotic signals will be generated in order to protect the organism by eliminating the damaged cells. Apoptosis signal-regulated kinase 1 (*ASK1*) is a mitogen-activated protein kinase, which is essential for the continuous activation of JNK and apoptosis (Tobiume et al., 2001). Cysteine-containing aspartate-specific proteases (Caspases) are a family of proteases that perform apoptosis in animals. The activation of caspase 3 (*CASP3*) indicates that apoptosis has entered an irreversible stage. Apoptosis mediated by ERS triggers a specific cascade of caspase 12, 9, and 3 (Morishima et al., 2002). All of these related genes (*GRP78*, *ATF6*, *IRE1*, *XBP1*, *PERK*, *eIF2α*, *ATF4*, *ASK1*, and *CASP3*) have been cloned, which makes studies of the UPR signaling pathway and apoptosis in *L. vannamei* available (Chen et al., 2012; Wang et al., 2013; Xu, Ruan & Shi, 2014; Yuan et al., 2016, 2017). However, the roles of UPR and apoptosis in cold stress have not been reported.

The hepatopancreas is a vital organ of the crustacean, having many important physiological functions, including those of immunity and digestion. The activity of alanine aminotransferase (ALT) in plasma can reflect damage to the hepatopancreas (Nyblom et al., 2004; Yan et al., 2016). Alkaline phosphatase (ALP) plays an important role in the immune system against pathogens, therefore, the activity of ALP in plasma can reflect the immunity level of shrimp. Glucose (Glu) is considered to be the main source of energy, while triglycerides (TG) and total cholesterol (TC) (the main components of lipids) can supply and store energy. Total protein (TP) is also able to provide energy and transport various metabolites.

In this study, the metabolite concentrations of plasma, the histology of the hepatopancreases, and gene expression related to UPR and cell apoptosis in the hepatopancreas and hemocytes of *L. vannamei* during acute cold-stress were analyzed. These results could provide useful information with which to investigate the physiological responses of shrimp in low temperatures.

## Materials and Methods

### Animals

The experimental *L. vannamei* (5.28 ± 0.50 g) were purchased from a commercial farm in Panyu (Guangdong, China). The shrimp were immediately transported to the lab and acclimated in 500 L air-pumped tanks filled with circulating diluted seawater for at least 4 days before the start of the experiments. During the acclimation stage, the water salinity and temperature in the tanks were consistent with those of the culture ponds (water salinity 5‰ and temperature 28 ± 1 °C). A commercial shrimp diet was fed twice per day.

### Acute cold-stress

A total of 45 healthy shrimp were randomly divided into three tanks. They were placed in an artificial climate incubator (Laifu) and the water temperature was decreased from 28 to 13 °C with a cooling rate of 2.5 °C/2 h and were maintained at 13 °C for 12 h.

### Sample collection

Hemolymph (250 uL) was obtained from the ventral sinus of the shrimp with a one mL sterile syringe containing an equal volume of ice-cold anticoagulant solution (27 mM trisodium citrate, 385 mM sodium chloride, 115 mM Glu, and pH 7.5). The hemocytes of two shrimp were pooled together as one sample, with three replicates at each treatment point (28, 23, 18, and 13 °C, and maintained in 13 °C for 12 h). After being centrifuged for 3 min at 3,000 rpm at 4 °C, the supernatant fluid was immediately stored at −80 °C for analysis of the plasma metabolite concentrations by an automatic biochemical analyzer (BS-200; Mindray, Shenzhen, China). The sediment (hemocytes) was collected, instantly frozen in liquid nitrogen, and then stored at −80 °C for gene expression analysis (Zhao et al., 2016). The hepatopancreas was dissected from the cephalothoraxes and washed with 0.85% pre-cooled sterilized normal saline at 4 °C. The whole hepatopancreas was collected for histological analysis and fixed with 4% paraformaldehyde (Biosharp, Hefei, China) for tissue fixation and stored at 4 °C for paraffin sections by Servicebio (Wuhan, China). Tissue slices were examined with microscopy (Nikon, Tokyo, Japan) and the Mshot Image Analysis System was used to photograph the stained sections. The hepatopancreas (40–50 mg) was then immediately frozen in liquid nitrogen for real time quantitative PCR and stored at −80 °C.

### RNA extraction and cDNA synthesis

Total RNA was extracted using RNAiso Plus reagent (Takara, Kusatsu, Shiga Prefecture, Japan) following the manufacturer’s protocol. RNA quality was assessed by electrophoresis on a 1.0% agarose gel and its concentration was tested by mySPEC (VWR, Radnor, PA, USA). The total RNA was purified and the first-strand cDNA was synthesized using ReverTra Ace^®^ qPCR RT Master Mix with gDNA Remover (TOYOBO, Osaka, Osaka Prefecture, Japan) according to the manufacturer’s instructions.

### Real-time quantitative PCR

The SYBR Green real-time PCR assays were carried out on a CFX Connect™ Real-Time System (BIO-RAD, Hercules, CA, USA) using THUNDERBIRD^®^ SYBR^®^ qPCR Mix without Rox (TOYOBO, Osaka, Osaka Prefecture, Japan). Specific primer sequences were designed based on the coding sequence of the target genes using Primer 6.0 software (Table 1). The real-time PCR program was set at 1 min for 95 °C, followed by 40 cycles of 95 °C for 15 s, 60 °C for 15 s, and 72 °C for 45 s, and by a final denaturation step of 95 °C for 10 s. Melting curves were obtained by increasing the temperature from 65 to 95 °C (0.5 °C/s) to denature the double-stranded DNA.

**Table 1:** Nucleotide sequences of the real-time PCR primers used in this study.

Primer names	Nucleotide sequences (5′–3′)
LvGRP78-F	TCATTGCCAACGACCAGGGT
LvGRP78-R	TCCGATGAGACGCTTGGCAT
LvPERK-F	TCCTGACATCATCATTATCATCTCC
LvPERK-R	TGAAGCTCATGCTCTCTGCCAATCC
LveIF2α-F	GGAACCTGTCGTTGTCATCAGAGTAG
LveIF2α-R	AGAAGCTCTCCAACATGCCGAATG
LvATF4-F	GCCACGATTCAAGATGCTGC
LvATF4-R	TCCTCCTCGTCCATGCCATA
LvATF6-F	CTGTTGGGACAAGGACCATAAGC
LvATF6-R	GAATTGTAGGTGTGGCAGCTGTTA
LvIRE1-F	TGGTGAGAAGCAGCTTGTGTTGG
LvIRE1-R	ACTGTTGATGAAGAGCCACTTGTAGC
LvXBP1-F	GTGGATCAGCAGTATCCCAACC
LvXBP1-R	TGCCAAGGCAGCTGTATTGA
LvCasp3-F	ACATTTCTGGGCGGAACACC
LvCasp3-R	GTGACACCCGTGCTTGTACA
LvASK1-F	GCTGTGTTGAAGTCCGAGGAGAAG
LvASK1-R	AGCCAAGCAACCAACTCCATATCG
LvActin-F	GACTACCTGATGAAGATCC
LvActin-R	TCGTTGCCGATGGTGATCA

## Results

### Plasma analysis

**Figure 1:**
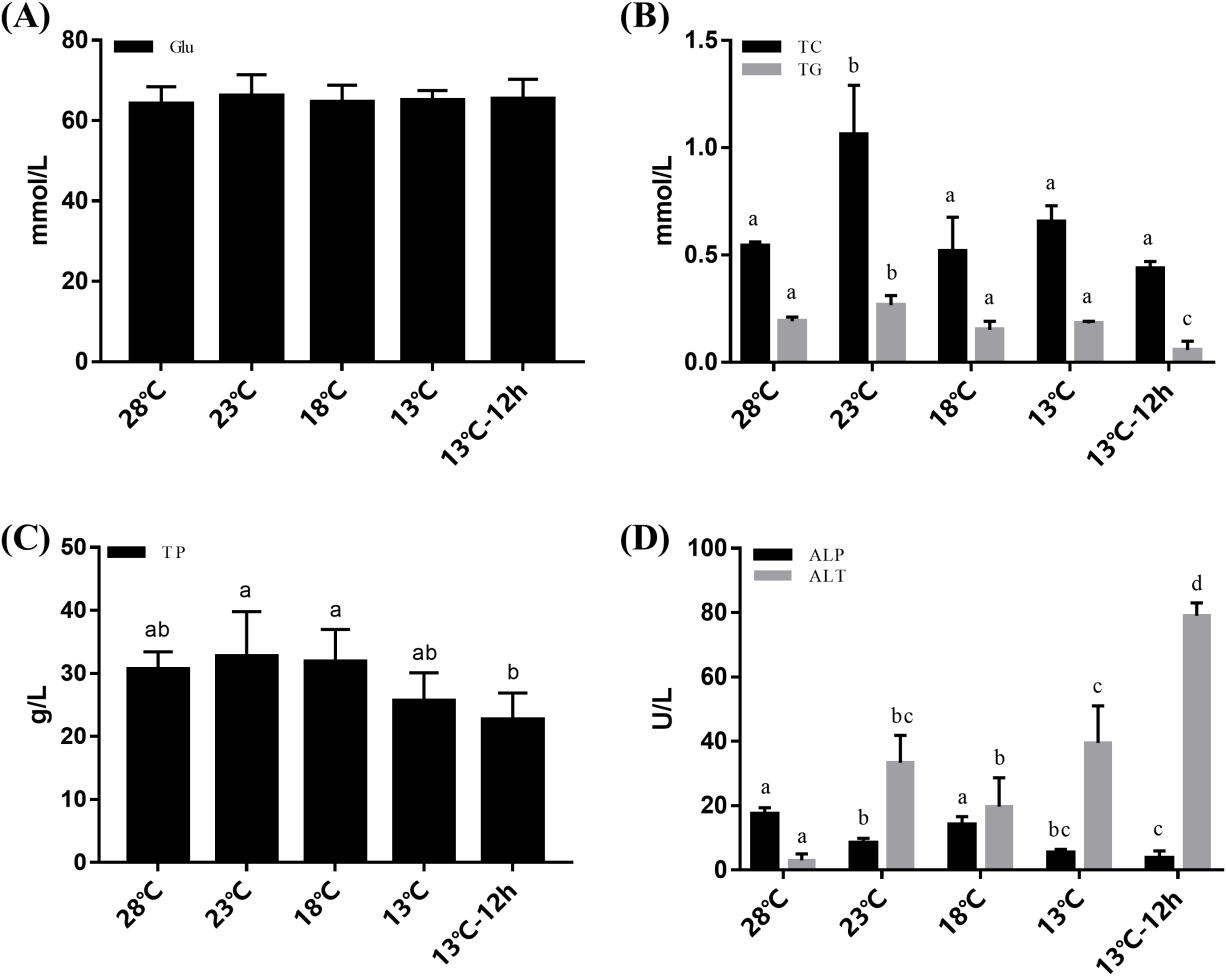
Plasma Glu, TG, TC, TP, ALP, and ALT in *L. vannamei* according to the acute cold stress. The glucose content (Glu) was shown in (A), triglyceride (TG), and total cholesterol (TC) content were shown in (B), total protein (TP) was shown in (C), and alkaline phosphatase (ALP) and alanine aminotransferase (ALT) activity were shown in (D) separately. The bars represent the mean ± S.D. (*n* = 3). Statistical significance was calculated by one-way ANOVA. Bars with different letters indicate statistical differences (*p* < 0.05).

The plasma of *L. vannamei* was measured at 28, 23, 18, 13, and 13 °C for 12 h, respectively. Changes in Glu content were not obvious during the cooling process (Fig. 1A). The concentrations of TC and TG increased after cooling and reached their peak at 23 °C. TC then returned to a normal level at 28 °C while TG significantly decreased at 13 °C for 12 h. The concentrations of TP remained stable from 28 °C to 13 °C and significantly decreased at 13 °C for 12 h (Fig. 1C). ALT activities in the plasma increased significantly after acute cold-stress. They reached their peak at 13 °C for 12 h, which was more than 26 times higher than that at 28 °C. However, ALP levels had the opposite reactions (Fig. 1D).

### Hepatopancreas histological analysis

**Figure 2:**
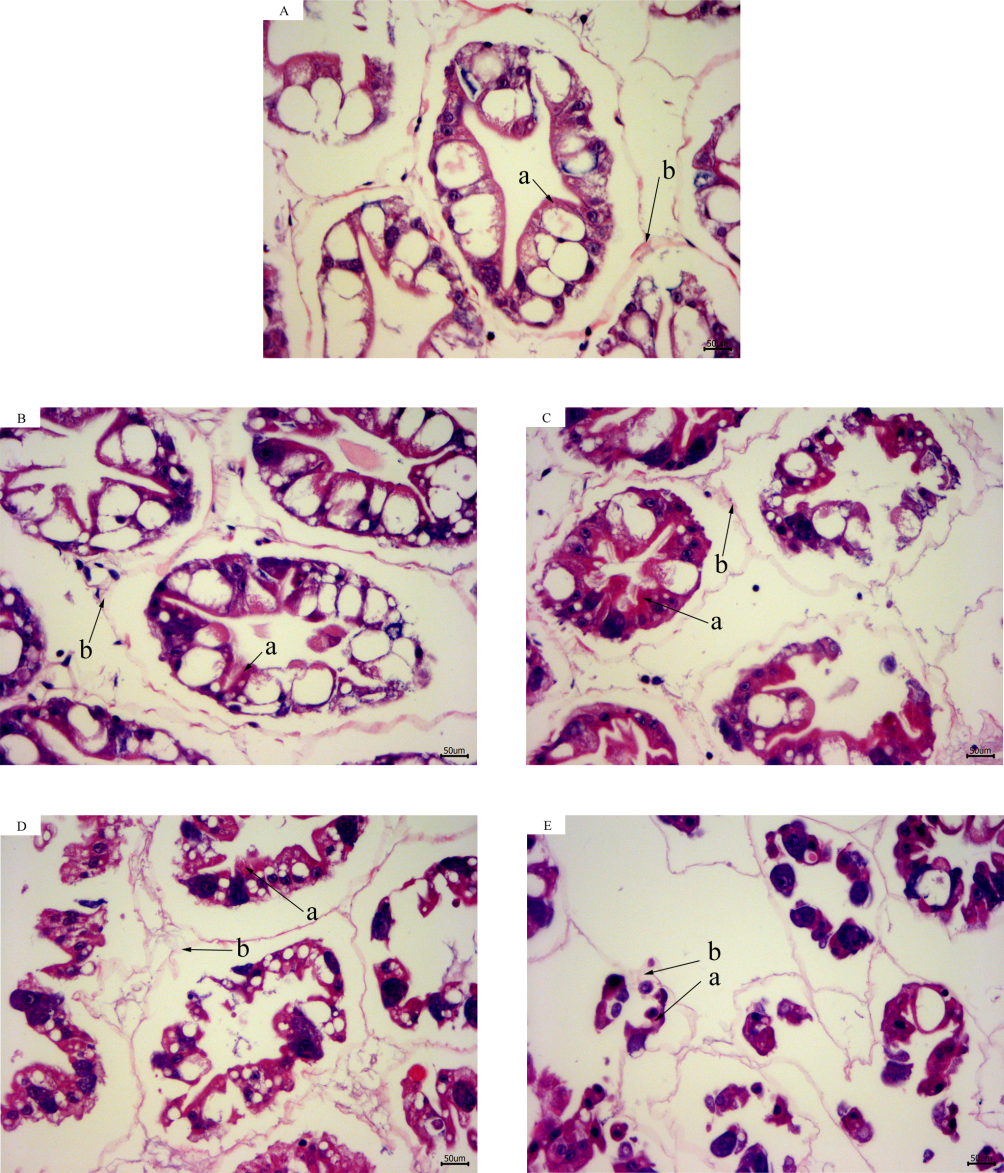
Hepatopancreas tissue structure (×400) of *L. vannamei* with HE dye after acute cold-stress. (A) 28 °C. (B) 23 °C. (C) 18 °C. (D) 13 °C. (E) 13 °C for 12 h. The letters in the figure indicated that: a, hepatocytes; b, basement membrane.

According to the results of hepatopancreas HE staining, the hepatocytes were arranged closely together with a clear cell gap at 28 °C. The boundaries of the hepatic tubules were blurred and the arrangement of hepatic cells became disordered with a decrease in temperature. At 13 °C for 12 h, most of the hepatic tubules were necrotic and the basement membranes were ruptured. Intact structures of the hepatic tubules were hardly observed (Fig. 2).

### Gene expression in hepatopancreas during acute cold-stress

**Figure 3:**
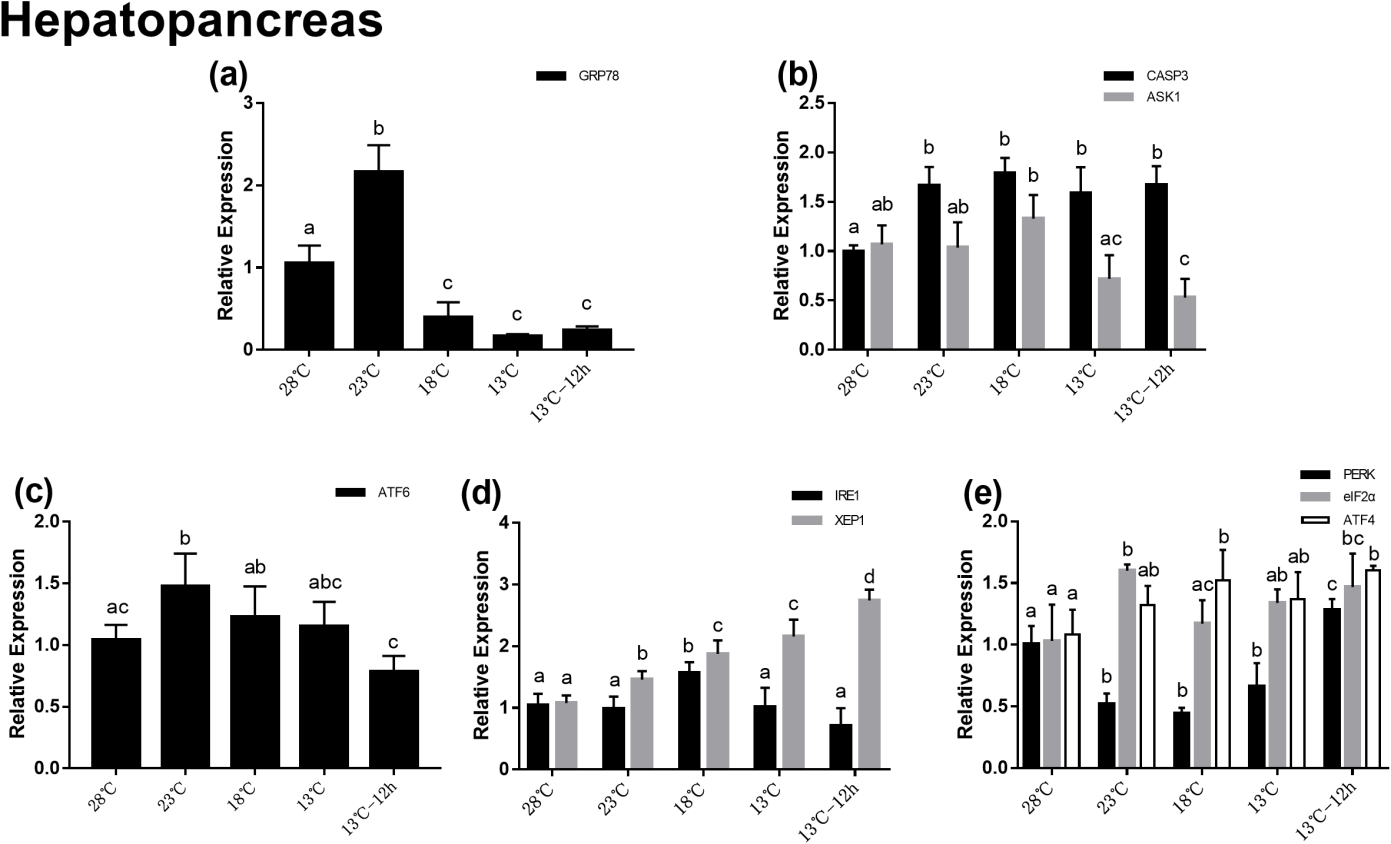
Relative expression of UPR and apoptosis related genes in hepatopancreas after acute cold-stress. Water temperature was decreased from 28 to 13 °C with a cooling rate of 2.5 °C/2 h and maintain in 13 °C for 12 h. The relative mRNA expression levels of GRP78 (A), apoptosis related genes (CASP3, ASK1) (B), ATF6 pathway (ATF6) (C), IRE1 pathway (IRE1, XBP1), (D) and PERK pathway (PERK, eIF2α, ATF4) (E) were compared with those at 28 °C. The bars represent the mean ± S.D. (*n* = 3). Statistical significance was calculated by one-way ANOVA. Bars with different letters indicate statistical differences (*p* < 0.05).

In the hepatopancreas, the relative mRNA expression of *GRP78* increased significantly at 23 °C, which was about twofolds at 28 °C, and then significantly decreased at 18 °C (Fig. 3A). In UPR, the expression of *ATF6* increased significantly with temperature changes from 28 to 23 °C, and then decreased gradually (Fig. 3C). In the *IRE1* pathway, there was no significant difference among the expression of *IRE1* at different temperatures, while the expression of *XBP1* significantly increased after acute cold-stress and its expression at 13 °C for 12 h was more than twofolds higher than that at 28 °C (Fig. 3D). In the *PERK* pathway, the expression of *PERK* decreased significantly at 23 °C, remained stable from 23 to 13 °C, and then significantly increased at 13 °C for 12 h. The expression of *eIF2α* and *ATF4* increased gradually. *PERK*, *eIF2α*, and *ATF4* appeared to be highly expressed at 13 °C for 12 h (1.28-, 1.47-, and 1.60-folds compared with that at 28 °C, respectively) (Fig. 3D). For genes related to apoptosis, the expression of *CASP3* increased significantly after acute cold-stress and remained at a stable level, which ranged from 1.58- to 1.79-folds higher than that at 28 °C, while the expression of *ASK1* significantly decreased after 13 °C and reached a minimum level at 13 °C for 12 h (Fig. 2B).

### Gene expression in hemocytes during acute cold-stress

**Figure 4:**
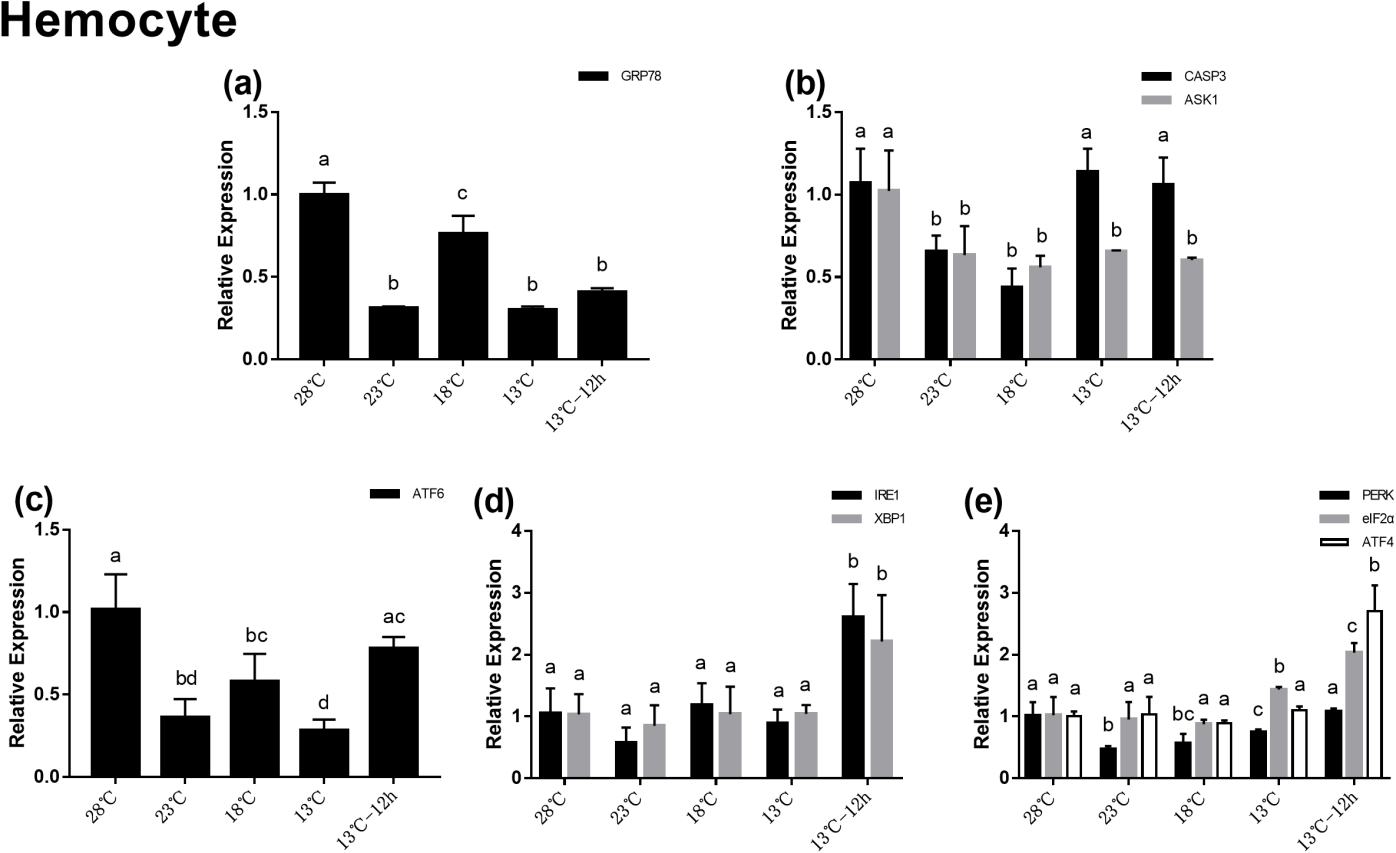
Relative expression of UPR and apoptosis related genes in hemocyte after acute cold-stress. Water temperature was decreased from 28 to 13 °C with a cooling rate of 2.5 °C/2 h and maintain in 13 °C for 12 h. The relative mRNA expression levels of GRP78 (A), apoptosis related genes (CASP3, ASK1) (B), ATF6 pathway (ATF6) (C), IRE1 pathway (IRE1, XBP1) (D), and PERK pathway (PERK, eIF2α, ATF4) (E) were compared with those at 28 °C. The bars represent mean± S.D. (*n* = 3). Statistical significance was calculated by one-way ANOVA. Bars with different letters indicate statistical differences (*p* < 0.05).

In the hemocytes, the expression of *GRP78* decreased significantly after acute cold-stress and then increased remarkably at 18 °C, which was more than twofolds higher than that at 23 °C (Fig. 4A). In UPR, the expression of *ATF6* showed the same trend as that of *GRP78* (Fig. 4C). In the *IRE1* pathway, the expression of *IRE1* and *XBP1* remained stable from 28 to 13 °C, and then significantly increased at 13 °C for 12 h (2.60- and 2.21-folds compared with those at 28 °C, respectively) (Fig. 4D). In the *PERK* pathway, the trends were the same as those in the hepatopancreas (Fig. 4E). For genes related to apoptosis, the expression of *CASP3* decreased at 23 °C and then increased above 13 °C. The expression of *CASP3* at 13 °C and 13 °C for 12 h were 1.14- and 1.06-folds compared with that at 28 °C, respectively and there were no significant differences among these at the three treatment point. The expression of *ASK1* significantly decreased after acute cold-stress and remained at a stable level, which ranged from 0.56- to 0.65-folds at 28 °C (Fig. 4B).

## Discussion

*Litopenaeus vannamei* is one of the most important aquaculture shrimps in the world. It is a warm-water shrimp that is sensitive to cold environments, therefore, low temperatures present a serious challenge to its survival, growth, and distribution (Peng et al., 2016; Cottin et al., 2016). In this study, the plasma metabolite concentrations, histological changes, and relative gene expression in the UPR signaling pathway and apoptosis genes induced by ERS in *L. vannamei* during acute cold-stress were investigated.

It has been well accepted that protein acts as the main energy source for shrimp (New, 1976; Zhang et al., 2006; Cuzon et al., 2010). And lipid was the main energy source of tilapia fish during long-time hypoxia stress (Li et al., 2018). In previous studies, the digestion and absorption of fat, and the protein pathways were significantly enhanced after cold adaptation by *L. vannamei* (He et al., 2018). In the current study, the results revealed that under low temperature stress, lipids (TG and TC, the major components of lipids) and proteins (TP) in the plasma significantly increased after acute cold-stress at 23 °C, while Glu did not change significantly. Thus, we speculated that protein and lipids are the main energy sources for *L. vannamei* during acute cold-stress.

It has been reported that the hepatopancreas was the main site for gluconeogenesis in decapod crustaceans (Reyes-Ramos et al., 2018; Berry et al., 2019). In this study, the results of HE staining revealed obvious damage to the hepatopancreas structure at 18 °C. Thus, combined with the hepatopancreas histology and plasma results, we speculated that the hepatopancreas facilitates gluconeogenesis to synthesize Glu from proteins and lipids, by which the shrimp are able to maintain their Glu demand under acute cold-stress. However, after the temperature dropped to 18 °C, the rupture of the hepatopancreas tubule resulted in the decrease of lipid and protein concentrations in the plasma. The regulation of energy metabolism after cold-stress in *L. vannamei* will require future study.

The present study has shown that ALP played a major role during the acute cold stress response in *Sparus aurata* and *L. vannamei*, this was probably because ALP may help protect the hepatopancreas and hemolymph from cold-stress damage (Mateus et al., 2017; Peng et al., 2018). The activity of ALT in plasma can reflect damage to the hepatopancreas. It has been shown that various forms of stress can cause an increase in plasma ALT activities in fish (Cho et al., 1994) and it is responsive to temperature change in fish (Costas et al., 2012). Environmental changes (e.g., cold shock and pH stress) have also been shown to cause damage to hepatopancreas cells, causing distension of the mitochondrial membrane, reduction or change of the hepatopancreas cells, and so on (Walker, Golden & Horst, 2010; Tao et al., 2016; Xu et al., 2018). In the present study, the ALP activity decreased to its lowest level and the ALT activity increased to its highest level at 13 °C for 12 h. The intact structure of the hepatic tubules can hardly be observed at these temperatures. Thus, combined with the histological and plasma results, we speculated that acute cold-stress could cause serious damage to the hepatopancreas of *L. vannamei* and therefore reduce their immunity.

Unfolded protein response restores the ER homeostasis by activating the *ATF6*, *IRE1*, and *PERK* pathways with *GRP78* being the key mechanism. Appropriate ERS can activate UPR to restore ER homeostasis and protect cells, however, if this imbalance exceeds its regulating ability, it will lead to apoptosis. In invertebrates, apoptosis is also an efficient defense system against stimuli harmful to organisms. Environmental stresses such as temperature stimulation, pH changes, and toxic substances can induce the apoptosis of shrimp cells and lead to cell death. In this study, the relative mRNA expression of *GRP78* in the hepatopancreas and hemocytes were significantly altered after acute cold-stress, which indicated that UPR was involved in this process. In previous studies, the differential expression of UPR and the apoptosis-related genes of the Pacific oyster were significantly enhanced in response to stress related to temperature change (Yang et al., 2017). In rainbow trout (Huang et al., 2018), protein processing in the ER pathway and lipid metabolism pathways were also involved in the heat stress response. The plasma and gene expression results in our study were consistent with these previous studies.

Moreover, in this study, the expression change trend of *ATF6* in the hepatopancreas and hemocytes were similar to that of *GRP78*. While the expression changes of *IRE1* and the *PERK* pathway related genes in the hepatopancreas and hemocytes were different from *GRP78*. Previous studies showed that *ATF6* was the first sensor to respond to UPR. But for *IRE1* and *PERK*, *GRP78* was a modulatory element, rather than a switch (Pincus et al., 2010; Gardner et al., 2013; Lewy, Grabowski & Bloom, 2017). These results was consistent with previous studies.

In addition, we found that the expression of some genes (*GRP78*, *ATF6*, and *CASP3*) significantly increased after acute cold-stress in the hepatopancreas, while in hemocytes the expression of these genes decreased significantly at 23 °C. This may be related to the damage of the hepatopancreas which is the hematopoietic organ of shrimp. Combined with plasma, histological, and apoptotic gene expression results, we found that the hepatopancreas was damaged after acute cold-stress, which resulted in the injury of hematopoietic function. Therefore, the number of hemocytes in the hemolymph of equal volume may be reduced (we extracted an equal volume of hemolymph at each time point). Thus, the expression of genes in the hemocyte may decrease. Previous study has also shown that the total hemocyte count decreased when *L. vannamei* was exposed to low water temperature (Fan, Wang & Wu, 2013). However, the specific mechanisms involved remain to be explored.

## Conclusions

In summary, *L. vannamei* may resist cold stress by utilizing proteins and lipids. Acute cold-stress causes damage to the hepatopancreas and reduces its immunity. All three UPR pathways are involved in the process of acute cold-stress. Moreover, compared with *IRE1* and the *PERK* pathway, *ATF6* was the first sensor to respond to UPR after acute cold-stress. Further research will focus on the significant changes in gene expression and their roles during cold stress.

## Supplemental Information

10.7717/peerj.7381/supp-1 Supplemental Information 1 The raw data of plasma metabolite concentrations for Fig. 1.

10.7717/peerj.7381/supp-2 Supplemental Information 2 The raw data of gene expression in hepatopancreas for Fig. 3.

10.7717/peerj.7381/supp-3 Supplemental Information 3 The raw data of gene expression in hemocytes for Fig. 4.

## Additional Information and Declarations

### Data Availability

The following information was supplied regarding data availability: The raw measurements are available in Table S1, Table S2, and Table S3. The raw data were used for statistical analysis about changes of plasma metabolites concentrations and relative mRNA expression during acute cold-stress.
